# Augmented human thermal discomfort in urban centers of the Arabian Peninsula

**DOI:** 10.1038/s41598-024-54766-7

**Published:** 2024-02-17

**Authors:** Safi Ullah, Abdullah Aldossary, Waheed Ullah, Sami G. Al-Ghamdi

**Affiliations:** 1 Environmental Science and Engineering Program, Biological and Environmental Science and Engineering Division, King Abdullah University of Science and Technology (KAUST), 23955-6900 Thuwal, Saudi Arabia; 2 KAUST Climate and Livability Initiative, King Abdullah University of Science and Technology (KAUST), 23955-6900 Thuwal, Saudi Arabia; 3 School of Computer, Data and Information Sciences, University of Wisconsin-Madison, Madison, WI 53715-1007 USA; 4 Defense and Security, Rabdan Academy, 114646 Abu Dhabi, United Arab Emirates

**Keywords:** Universal Thermal Climate Index (UTCI), ERA5-HEAT, Thermal discomfort, Urban centers, Arabian Peninsula (AP), Climate sciences, Atmospheric science, Climate change, Public health

## Abstract

Anthropogenic climate change has amplified human thermal discomfort in urban environments. Despite the considerable risks posed to public health, there is a lack of comprehensive research, evaluating the spatiotemporal changes in human thermal discomfort and its characteristics in hot-hyper arid regions, such as the Arabian Peninsula (AP). The current study analyzes spatiotemporal changes in human thermal discomfort categories and their characteristics in AP, using the newly developed high-resolution gridded ERA5-HEAT (Human thErmAl comforT) dataset for the period 1979–2022. In addition, the study assesses the interplay between the Universal Thermal Climate Index (UTCI) and El Niño-Southern Oscillation (ENSO) indices for the study period. The results reveal a significant increase in human thermal discomfort and its characteristics, with higher spatial variability in the AP region. The major urban centers in the southwestern, central, and southeastern parts of AP have experienced significant increases in human thermal discomfort (0.4–0.8 °C), with higher frequency and intensity of thermal stress during the study period. The temporal distribution demonstrates a linear increase in UTCI indices and their frequencies and intensities, particularly from 1998 onward, signifying a transition towards a hotter climate characterized by frequent, intense, and prolonged heat stress conditions. Moreover, the UTCI and ENSO indices exhibit a dipole pattern of correlation with a positive (negative) pattern in the southwestern (eastern parts) of AP. The study’s findings suggest that policymakers and urban planners need to prioritize public health and well-being in AP’s urban areas, especially for vulnerable groups, by implementing climate change adaptation and mitigation strategies, and carefully designing future cities to mitigate the effects of heat stress.

## Introduction

Human thermal comfort refers to the state where individuals feel satisfied or comfortable with their surrounding thermal environment^1,2^. The intertwining impacts of climate change and rapid urbanization have significantly altered the thermal dynamics of urban environments, posing substantial challenges related to heat stress and thermal comfort^3,4^. As global temperatures continue to rise, urban areas experience amplified heat stress or discomfort due to urban heat island (UHI) effects, characterized by increased temperature and altered urban microclimates^5,6^. In UHI, urban areas are relatively experiencing higher temperatures than their surrounding rural areas, which is influenced by two main factors, i.e., impermeable surfaces and albedo. The proliferation of impermeable surfaces, such as asphalt and concrete, absorb and radiate heat, creating localized warming and heat stress^6^. Albedo, on the other hand, is a measure of how much sunlight is reflected by a surface, and thus low albedo surfaces or dark surfaces, like asphalt and roofs, absorb more sunlight and heat up, affecting the overall thermal comfort level in urban areas^7^. Urbanization also leads to the loss of green spaces, reducing the cooling effect of vegetation and exacerbating the heat stress and thermal discomfort in urban areas^8^. The resultant human thermal discomfort poses a multifaceted challenge, affecting both indoor and outdoor urban environments. The increase in human thermal discomfort also leads to adverse impacts on human health, labor, ecosystems, and the overall economy^9–12^. The increasing tendency of human thermal discomfort, with profound and multifaceted consequences, underscores the need for both mitigation and adaptation strategies. Timely and accurate assessment of human thermal stress is critical for determining the magnitude and extent of human thermal discomfort, identifying thermal stress hotspots, and mitigating potential effects on natural and built environments^13–15^.

To date, various thermal stress indices have been developed and employed to quantify human thermal stress, including apparent temperature (AT), discomfort index (DI), heat index (HI), humidity index (Humidex), wet-bulb temperature (WBT), wet-bulb-globe temperature (WBGT), the universal thermal climate index (UTCI), etc. All these indices provide insights into the complex interplay between human comfort and environmental factors. According to Yan et al.^4,12^, thermal indices are designed to quantify the combined effects of various environmental parameters on the human body. Among the above-mentioned indices, UTCI is a widely accepted and reliable indicator of thermal comfort, accounting for both physiological and environmental factors^13,16,17^. It is an important index used for a comprehensive assessment of human thermal discomfort, by integrating multiple bio-meteorological factors, such as air temperature, relative humidity, wind speed, solar and thermal radiation, human clothing type, and physical activity level^12,18–20^. The robust and comprehensive assessments of human thermal stress utilizing the UTCI can empower decision-makers to take necessary actions and formulate policy interventions aimed at reducing the adverse impacts of climate change-induced thermal stress in vulnerable regions^21,22^.

UTCI is currently being used in many parts of the world, to assess changes in human thermal discomfort and other related factors^17^. A review study on the use of UTCI found that 60% of the UTCI applications were in outdoor thermal comfort assessment and urban climate and planning studies, while the other 40% of UTCI applications were on climate change-related health impacts, bioclimates, meteorological analyses, climate change research, and tourism^23^. In recent years, UTCI has been extensively employed to assess heat-related health risks and identify thermal bioclimatic variability in different parts of Europe^15,22,24–30^. Similarly, some studies have utilized UTCI to assess observed spatiotemporal changes in human thermal stress in different regions of the Asian continent^13,31–37^. UTCI is also employed in southern and northern American countries to evaluate the level of human thermal comfort and the potential impacts of heat stress under the current global warming trend^16,38–41^. Despite being an important index for assessing human thermal comfort, UTCI's application is limited in certain parts of the world, including the Arabian Peninsula (AP). Regions such as AP, which experience extreme heat stress, require urgent studies to assess and measure human thermal discomfort and understand its underlying mechanisms.

AP is characterized by its arid and hot climate^42^. The region has experienced pronounced warming with frequent and intense heat extremes that will continue in the future with more catastrophic implications^43–46^. To date, a diverse suite of indices has been used to assess observed and projected spatiotemporal changes in human thermal stress in the AP and neighboring regions. For instance, Christidis et al.^47^ used high-risk warming and high-risk days indices to estimate the likelihood of extremely hot days (≥ 50 °C) and heat-related mortalities in the Mediterranean and Middle East regions. Whereas Hajat et al.^48^ applied the AT index to quantify the current and future trends in heat-related mortality in the Middle East and North Africa (MENA) region. In another study, Safieddine et al.^43^ used a WBT index to analyze current and future human heat stress in the AP, while Kang et al.^49^ applied WBT and dry-bulb temperature indices to estimate future heat stress during the Muslim Pilgrimage (Hajj) season. Similarly, Ahmadalipour and Moradkhani^50^ employed a WBT index to anticipate future heat stress conditions in the MENA region. Some studies also assessed observed changes in human thermal stress in Saudi Arabia, such as Dasari et al.^51^ used the DI to analyze the trends and variability of outdoor thermal discomfort, whereas Al-Bouwarthan et al.^52^ employed WBGT to determine the exposure of construction workers to heat stress.

El Niño-Southern Oscillation (ENSO) is a recurring climate pattern involving changes in the temperature of surface waters in the central and eastern tropical Pacific Ocean, which includes three phases: El Niño, La Niña, and neutral^53,54^. The El Niño phase is associated with the anomalous warm surface water accumulation in the central and eastern Pacific Ocean, the La Niña phase conversely represents the contrary with anomalously cool water in the central and eastern Pacific Ocean^55,56^. The neutral phase occurs in between El Niño and La Niña events, with sea surface temperatures (SST) and atmospheric conditions returning to near-average levels. Both El Niño and La Niña phases interact with the overlying atmospheric circulation, which leads to various global impacts such as changes in rainfall patterns, droughts, and flooding. ENSO indices' correlations with monthly mean global precipitation and surface temperature reveal the ENSO3.4 and relative ENSO3.4 indices as the strongest for capturing ENSO's global precipitation impact. Conversely, the Nino4 and Nino3.4 indices excel in capturing ENSO's influence on surface temperature, with the Modoki index being the weakest^57^. Researchers in the past have identified different geographical regions in the Pacific Ocean where the SST anomalies as a precursor to ENSO evolution with a broader global implication. The notable indices include ENSO3.0, 3.4, and 4.0. ENSO3.0 (5N–5S, 150W–90W) was initially considered the key region for monitoring and predicting the ENSO and coupled ocean–atmosphere interactions, but the scientists discovered that this area lies further west, and hence ENSO3.4 became more favorable^53,57^. The ENSO3.4 index (5N–5S, 170W–120W) uses a 5-month running mean, and El Niño or La- Niña events are defined when the Niño 3.4 SSTs exceed +/− 0.4 °C for a period of six months or more. ENSO4.0 (5N–5S, 160E–150W) is in the central Pacific Ocean and exhibits less variance than the rest of the indices and monitors the eastward propagation of the ENSO.

Though the previously mentioned studies focused on evaluating human thermal stress in the MENA region using various indices, there is very limited literature available regarding the utilization of UTCI for assessing human thermal stress in the AP region. To date, only two studies applied UTCI for human thermal assessment in the AP region^58,59^; however, these studies were limited to a specific country, i.e., United Arab Emirates (UAE) and Saudi Arabia, and did not cover the whole AP region. Moreover, none of these studies have used the newly developed high-resolution gridded ERA5-HEAT (Human thErmAl comforT) dataset, which includes multiple human thermal stress indices, including UTCI^60^. Given these limitations, the present study employs ERA5-HEAT data to evaluate observed spatiotemporal changes in human thermal stress based on UTCI from 1979 to 2022. In addition, the study explores the interplay between the UTCI indices and the El Niño-Southern Oscillation (ENSO) indices in the AP region. The study findings provide valuable insights for developing effective measures to mitigate human thermal stress and its associated adverse consequences in the region.

## Study area

**Figure 1:**
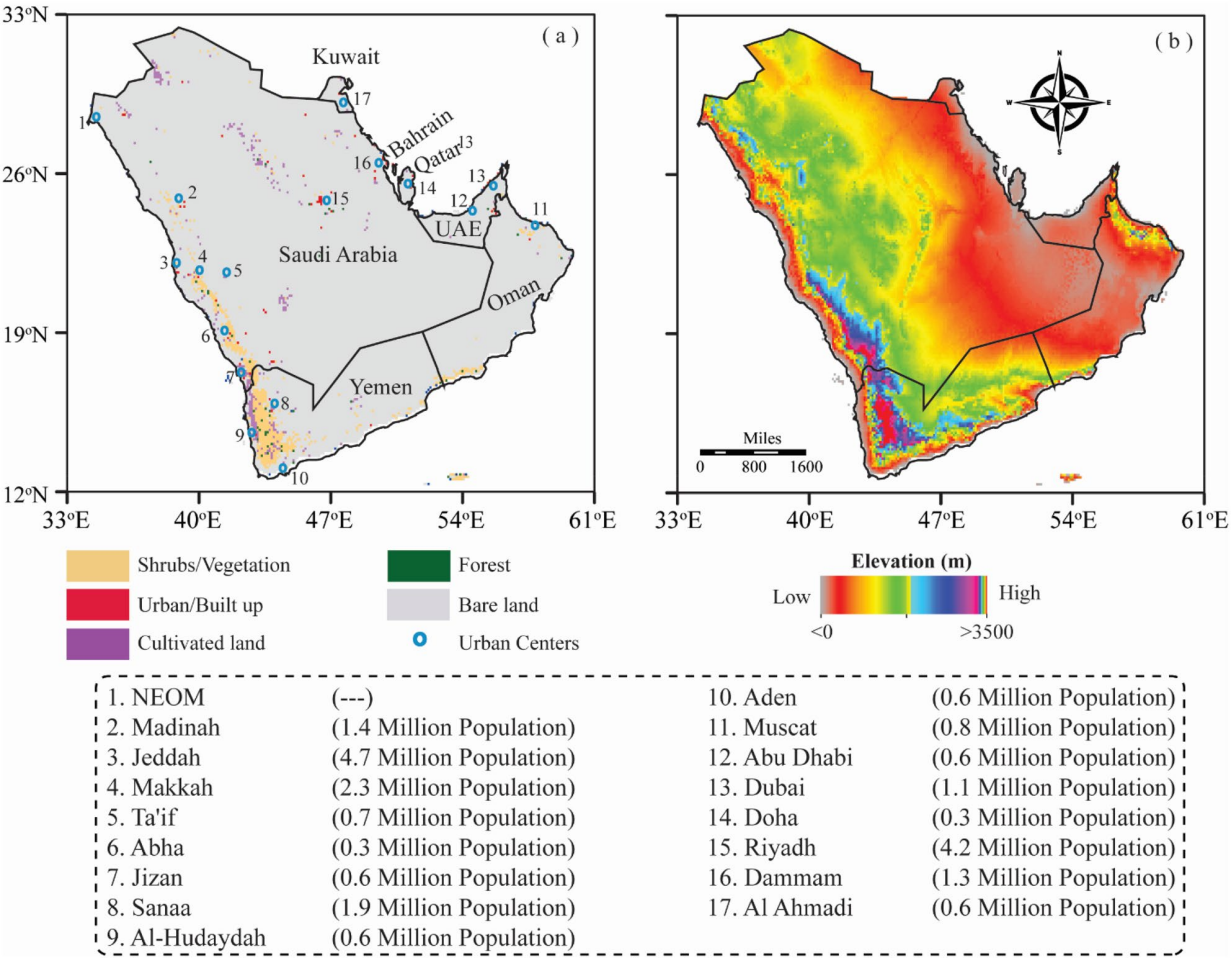
Topographic map of the Arabian Peninsula (AP); (**a**) land cover classes, the AP member countries, the major urban centers and their respective population, and (**b**) digital elevation model (DEM) (This fgure was created in ArcGIS 10.8).

Arabian Peninsula (AP) is a vast and significant geographic region located in Southwest Asia, with geographical coordinates of 12°–32° North latitudes and 34°–60° East longitudes (Fig. 1). AP is the world's largest peninsula, with an estimated area of 3.2 million km^2^, covering seven countries: Bahrain, Kuwait, Oman, Qatar, Saudi Arabia, the UAE, and Yemen (Fig. 1a). The peninsula is surrounded by major water bodies from multiple sides: the Persian Gulf to the northeast, the Red Sea to the west, the Gulf of Aden to the southwest, the Arabian Sea to the south, and the Gulf of Oman to the southeast. The region has diverse topography, including deserts, mountains, plains, and coastal regions. The Rub'al Khali (Empty Quarter) is one of the largest continuous sand deserts in the world, covering an estimated area of 650,000 km^2^, which is a significant portion of the peninsula’s southern region. The Hijaz and Asir mountain ranges run along the western coast, with some peaks exceeding 3000 m, and they are the highest elevated points in the peninsula (Fig. 1b). The climate of AP is arid to semi-arid, with high annual temperatures and limited precipitation, which make it one of the most vulnerable regions to climate change. The region exhibits spatiotemporal variability in temperature distribution, with a relatively colder climate in the northern parts, a hotter climate in the southern parts, and a moderate climate in the coastal areas. Generally, summers are extremely hot, with maximum temperature ranging from 40 to 50 °C during the daytime, whereas winters are milder, with minimum temperature varying from 8.57 to 10 °C during the nighttime. In AP, precipitation is generally low, with most of the areas receiving less than 100 mm of rainfall annually.

## Data and methods

### Data

The ERA5 is the fifth and latest version of the European Centre for Medium-Range Weather Forecasts (ECMWF) reanalysis gridded products. Among the ECMWF reanalysis datasets, the ERA5-HEAT, developed by Di Napoli et al.^60^, is a comprehensive gridded climatic data consisting of various thermal comfort indices, including UTCI. The ERA5-HEAT data are validated against global synoptic stations data, where the UTCI dataset exhibits robust performance, with correlation coefficients exceeding 0.6 (0.80 ± 0.13) at most of the global stations and ranging from 0.9 to 1 in the AP region. Similarly, the root-mean-square error averages 5.2 ± 2.5 °C globally and varies from 0 to 5 °C in the AP region. ERA5-HEAT is an hourly dataset, with a horizontal resolution of 0.25° × 0.25°, spanning from 1979 to the present date. In this study, we calculated the daily minimum (UTCI_min_), maximum (UTCI_max_), and mean (UTCI_mean_) UTCI values to assess spatiotemporal changes in daily human thermal discomfort in the AP region for the period 1979–2022. Here the “human thermal discomfort” refers to the feeling or sensation of being too hot or too cold, due to the interaction between environmental conditions and the human body's temperature regulation^20^. The ERA5-HEAT data are freely available for public use on the Copernicus Climate Data Store (CDS) portal, which is a part of the Copernicus Climate Change Service (C3S), regulated by ECMWF (https://cds.climate.copernicus.eu/cdsapp#!/dataset/derived-utci-historical?tab=overview).

To explore the relationships between the ENSO and UTCI indices in AP, the study also used the monthly time series of three different types of ENSO indices, including ENSO3.0, ENSO3.4, and ENSO4.0. The use of three different ENSO indices aims to understand the complex nature of ENSO's effects, and its regional variability, providing a holistic insight into the diverse ways ENSO influences thermal comfort in the peninsula. The ENSO indices data are obtained from the National Center for Atmospheric Research (NCAR) for the period 1979–2022 (https://psl.noaa.gov/data/climateindices/list/).

### Methods

#### Calculation of UTCI and its classes

To date, more than 100 different indices have been developed to thoroughly assess human thermal discomfort, including the UTCI. In this study, we preferred the use of UTCI over other indices because it is one of the widely used and reliable thermal comfort indicators that integrate both physiological and environmental factors^13,16,17^. Moreover, it is used as a biometeorology index to characterize and predict human thermal sensation based on a combination of multiple meteorological variables^61^. As a human heat exchange model, it is often applied to study the interplay between human health and meteorological factors^12,18–20^. UTCI is based on an operational procedure that employs a sixth-order polynomial regression approximation equation^62^. In ERA5-HEAT, the UTCI is computed using 2-m air temperature (T_a_), mean radiant temperature (MRT), humidity (RH), and wind speed (w) from the ECMWF ERA5 reanalysis, whereas the radiation element was used to calculate the MRT^60^. The mathematical equation of UTCI is as follows (Eq. 1):

1 $$UTCI \left({T}_{a}, MRT, w, RH\right)= {T}_{a}+offset ({T}_{a},MRT, w, RH)$$

where *T*_*a*_ is the 2-m air temperature (°C), *MRT* is the mean radiant temperature (°C), *w* is the 10-m wind speed (m/s), and *RH* is the relative humidity (%). Despite its effectiveness, the UTCI calculation has some limitations, including the requirement of the Ta within the range of − 50 to 50 °C, MRT-Ta within the range of − 30 to 70 °C, and 10-m wind speed should not exceed 17 m/s. More details about the processing and computational procedures of the EAR5-HEAT data are provided by Di Napoli et al.^60^. The AP climate is within these ranges; therefore, the application of the UTCI is suitable in this region.

According to the human body’s thermal physiological response corresponding to the model’s comfort standards, the UTCI values are typically categorized into ten thermal stress levels^13,21^. Further insights into these UTCI stress categories and their associated physiological conditions can be found in the work of Bröde et al.^62^. Given the prevalent hot tropical climate of the peninsula and the substantial warming observed in recent decades^63,64^, we considered only six thermal stress categories, primarily representing hot conditions in the study region. The selected human thermal stress categories along with their corresponding physiological conditions are presented in Table 1.

**Table 1:** UTCI categories and their corresponding physiological conditions.

S. no	UTCI (°C) range	Stress category	Thermal perception
1	0–9	Slight cold stress	Cold
2	9–26	No thermal stress	Comfortable
3	26–32	Moderate heat stress	Warm
4	32–38	Strong heat stress	Hot
5	38–46	Very strong heat stress	Hottish
6	> 46	Extreme heat stress	Torrid

#### ENSO indices

The three ENSO indices include ENSO3.0, 3.4, and 4.0, which are a representation of the Pacific SST evolution with circumglobal circulations, indirectly affecting the AP's temperature variability^65–67^. These indices used here represent an optimal approach to map different phases of the Pacific SST and its relationship with the UTCI indices in the AP.

#### Statistical analysis

The study employed various statistical techniques to determine spatiotemporal changes in human thermal discomfort in the study region. The non-parametric Sen’s slope estimator (SSE)^68^ test was used to estimate the slope of the trend, while the modified Mann–Kendall (m-MK)^69^ was employed to determine the statistical significance of the trend in UTCI indices and their stress categories during the study period. We preferred these non-parametric tests over other statistical tests as they are simple and robust against outliers and missing values in a time series^70,71^. Furthermore, these tests are less sensitive to abrupt breaks in a time series and do not require data normalization^72,73^. More details about SSE and m-MK tests can be found in recent studies^74,75^. Furthermore, we explored the relationship between ENSO types and UTCI indices using Pearson's correlation test. The 2-tailed Student *t*-test was employed to determine the statistical significance of correlations at the 95% confidence level. We used CDO (Climate Data Operators) version 2.2.0, Matlab (MATrix LABoratory) version R2022b, ArcGIS version 10.8, and GrADS (Grid Analysis and Display System) version grads-2.2.1 for data pre-processing, data analyses, and data visualization.

## Results

### Spatiotemporal changes in UTCI indices

**Figure 2:**
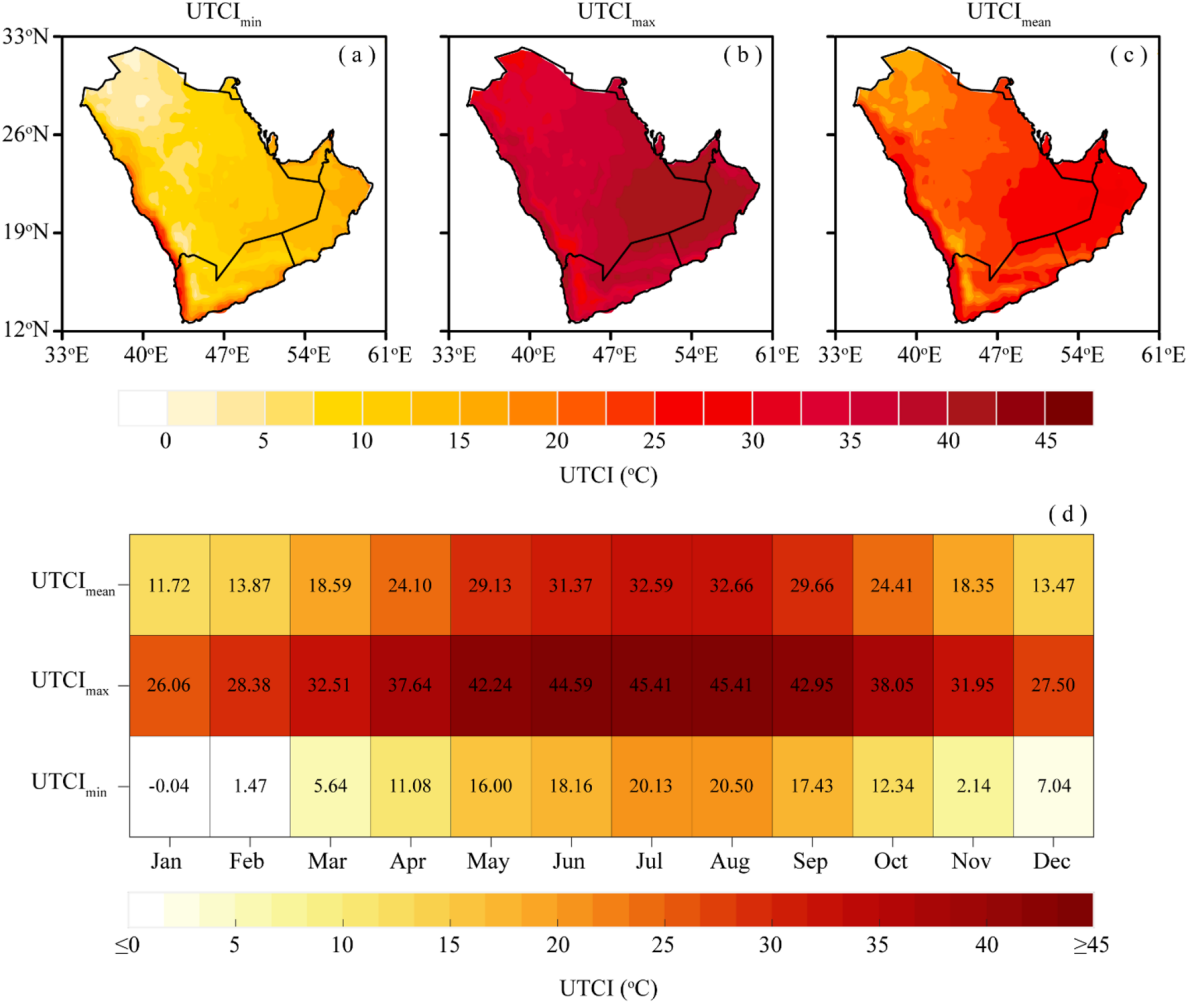
Long-term climatological means of UTCI indices over AP during 1979–2022; (**a**–**c**) spatial climatological means of UTCI_min_, UTCI_max_, and UTCI_mean_, respectively, and (**d**) monthly means of UTCI_min_, UTCI_max_, and UTCI_mean_ (In this fgure, panels (**a**–**c**) were created in GrADS 2.2.1, while panel (**d**) was created Matlab R2022b).

Figure 2 shows the long-term spatiotemporal changes and trends in UTCI_min_, UTCI_max_, and UTCI_mean_ over AP during 1979–2022. As seen in Fig. 2a–c, the spatial distribution of long-term climatological means of UTCI indices exhibits large variability in AP, with the highest magnitude (20–45 °C) in the southeastern part and southwestern coastal belt, indicating these parts of the AP had experienced considerable thermal stress in recent decades. The southeastern part of the AP is located along the border intersection of Oman, Saudi Arabia, UAE, and Yemen, having a hyper-arid climate and desert topography. Moreover, the southwestern belt of AP includes the major cities of Jeddah, Makkah, Al-Hudaydah, and Aden. Moreover, the central and eastern parts of the AP, mostly encompassing the metropolitan cities of Riyadh, Dammam, Doha, and Al Ahmadi, tend to experience UTCI magnitudes within the range of 10–35 °C. While the northern and northwestern parts experienced the lowest climatological intensity of UTCI indices (0–25 °C) during the study period. In terms of UTCI indices, the long-term climatological magnitude of UTCI_min_, UTCI_max_, and UTCI_mean_ ranges from 0 to 30 °C, 25 to 45 °C, and 15 to 35 °C, respectively.

As shown in Fig. 2d, the overall intensities of monthly UTCI_min_, UTCI_max_, and UTCI_mean_ range from − 0.04 to 20.50 °C, 26.06 to 45.41 °C, and 11.72 to 32.66 °C, respectively. The monthly analyses further show that July and August have the warmest thermal conditions, followed by June, September, and May with observed maximum intensities of 45.41 °C, 44.49 °C, 42.95 °C, and 42.24 °C, respectively. In contrast, January has the coldest thermal conditions, followed by February, March, and December with minimum UTCI intensities of − 0.04 °C, 1.47 °C, 5.64 °C, and 7.04 °C, respectively. These results indicate that the AP is under the strong influence of thermal stress during the summer months.

**Figure 3:**
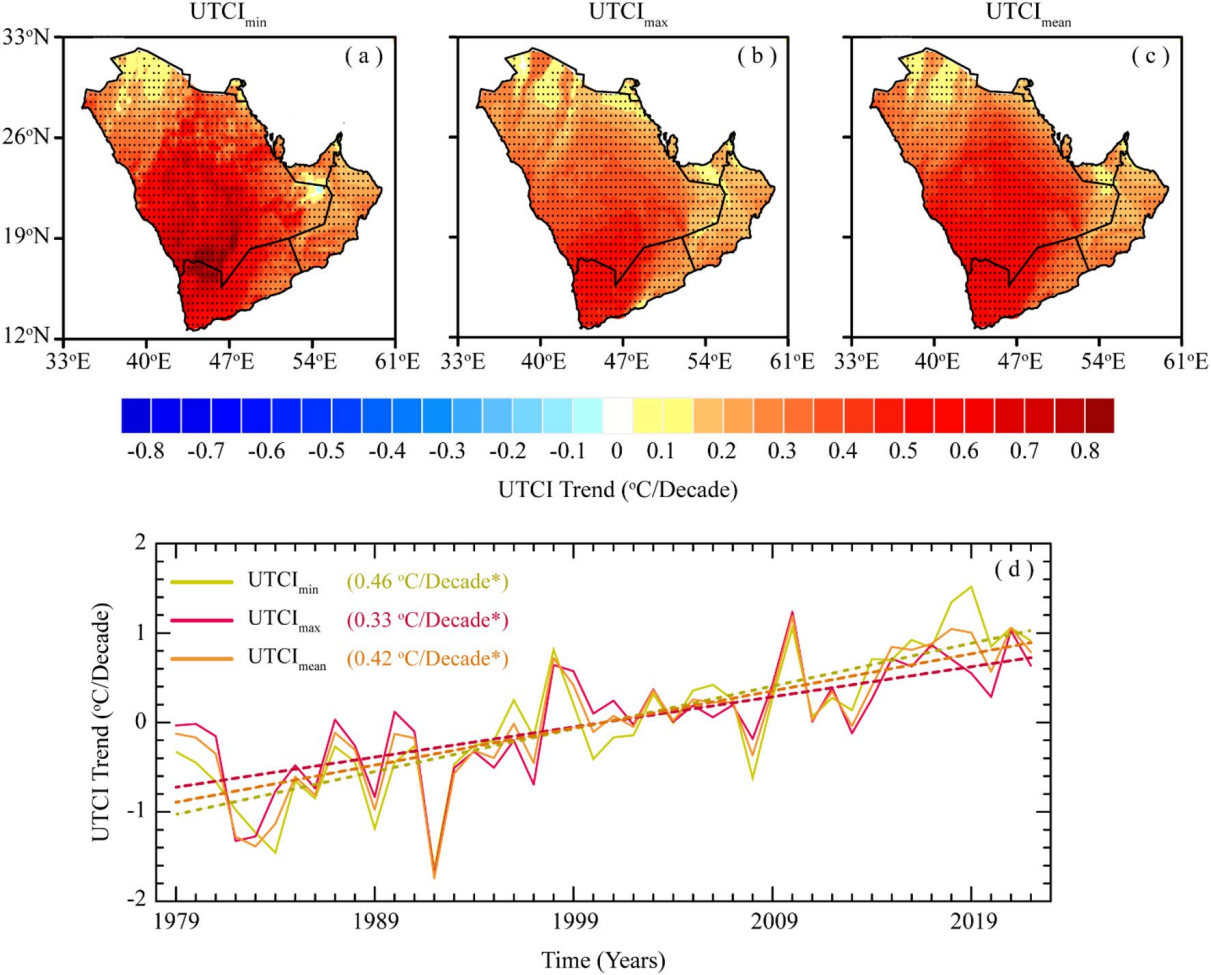
Long-term trends of UTCI indices over AP during 1979–2022; (**a**–**c**) spatial trends of UTCI_min_, UTCI_max_, and UTCI_mean_, respectively, and (**d**) temporal trends of UTCI_min_, UTCI_max_, and UTCI_mean_. The black dots in figures (**a**–**c**) indicate that the trend is statistically significant at the 0.05 significance level. The values in Figure (**d**) represent the monotonic trends of UTCI indices, while the asterisk (*) indicates that the trend is statistically significant at the 0.05 significance level (In this fgure, panels (**a**–**c**) were created in GrADS 2.2.1, while panel (**d**) was created Matlab R2022b).

The spatiotemporal trend in UTCI indices over the AP during 1979–2022 is presented in Fig. 3. The rising trend of UTCI indices shows a significant increase in thermal stress with higher spatial variability during the study period (Fig. 3a–c). The southwestern parts of AP, consisting of mountains and coastlines, exhibit a substantial increase of 0.6–0.8 °C/decade in UTCI indices. The central regions of AP also exhibit a notable warming trend in UTCI indices, ranging from 0.4 to 0.6 °C/decade. It is worth mentioning that both the southwestern and central parts of AP comprised some of the several populated cities, including Al-Hudaydah, Sanaa, Abha, Jeddah, Jizan, Makkah, Madinah, and Riyadh. The results further show that the northeastern and southeastern parts of AP experienced a relatively least increasing trend of 0.10–0.30 °C/decade in UTCI indices, which indicates that the stated areas have observed the lowest heat stress during the study period. These include the junction of the Saudi–Yemen–Oman border, and some major coastal metropolitan cities, such as Dammam, Dubai, Abu Dhabi, Muscat, Doha, and Al Ahmadi. Overall, the magnitude and spatial extent of the warming trend are larger in UTCI_min_, followed by UTCI_mean_ and UTCI_max_. As shown in Fig. 3d, the long-term temporal trend in annual anomalies of UTCI indices exhibits a linear increase during the study period. However, this increasing trend became more consistent during 1998 and onward, indicating a transition toward intensified regional climate warming and the emergence of frequent and intense heat extremes in AP. The monotonic trend results reveal that the UTCI_min_ exhibits the highest significant increasing trend, followed by UTCI_mean_ and UTCI_max_ at the rates of 0.46 °C, 0.42 °C, and 0.33 °C per decade, respectively.

### Spatiotemporal changes in UTCI stress categories

**Figure 4:**
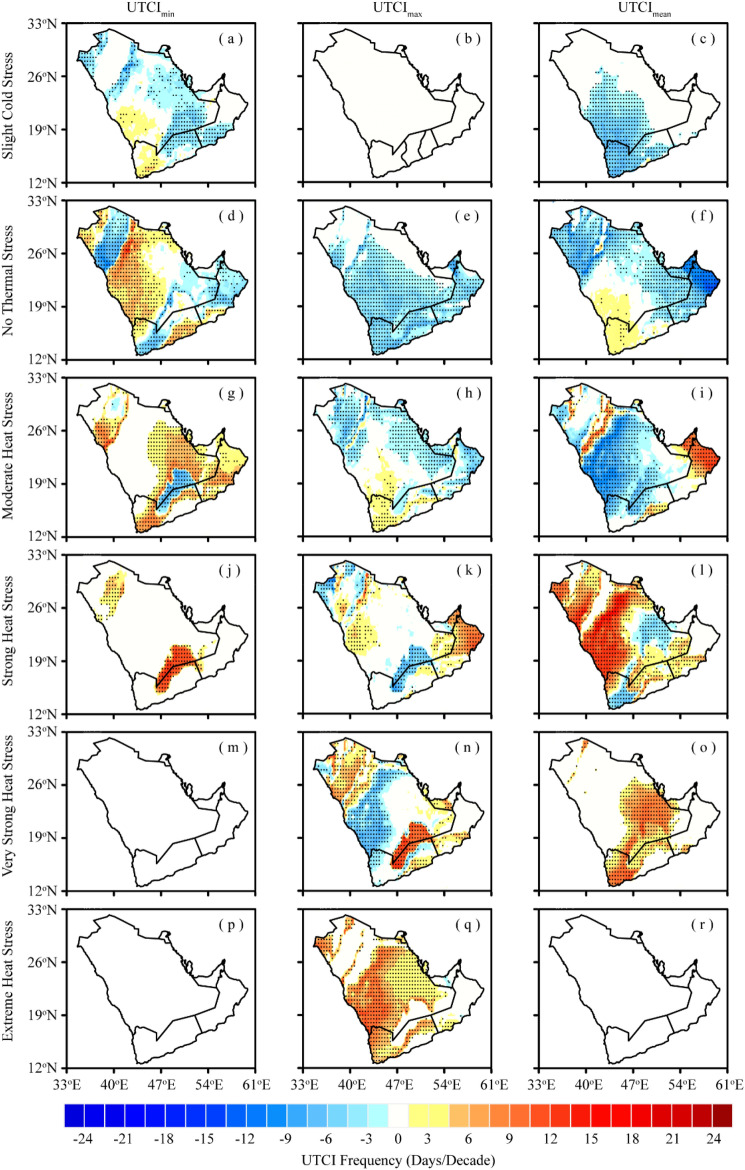
Spatial trends in the frequency of UTCI stress categories over AP during 1979–2022; (**a**–**c**) Slight cold stress, (**d**–**f**), No thermal stress, (**g**–**i**) Moderate heat stress, (**j**–**l**), Strong heat stress, (**m**–**o**) Very strong heat stress, and (**p**–**r**) Extreme heat stress. The black dots indicate that the trend is statistically significant at the 0.05 significance level (This fgure was created in GrADS 2.2.1).

Figure 4 depicts the spatial trend of the frequency of UTCI stress categories over AP during 1979–2022. Based on UTCI_min_, and UTCI_mean_ (Fig. 4a,c), a substantial reduction in the number of “slight cold stress” days is evident across most areas of the AP (–1 to –9 days/decade), except for the southern territories where a marginal increase in UTCI_min_-based “slight cold stress” days is observed (1–4.5 days/decade). It is noteworthy that UTCI_max_ does not signify any instances of “slight cold stress” days during the study period (Fig. 4b), which can be attributed to the consistently higher daily maximum temperatures in the AP that do not fall within the range of “slight cold stress” category throughout the year. Furthermore, the frequency of days falling into the “no thermal stress” category reveals an augmentation of 3–15 days/decade in the southwestern, central, and northwestern regions (Fig. 4d–f). Nonetheless, the magnitude and spatial extent of this increasing trend are more pronounced in UTCI_min_ than in UTCI_max_ and UTCI_mean_. Conversely, the remaining regions of the AP have experienced a decreasing trend in the “no thermal stress” days, with a more notable decline (–12 to –15 days/decade) in UTCI_mean_ observed along the Oman-UAE border (Fig. 4f).

As presented in Fig. 4g–i, the count of days characterized by “moderate heat stress” has risen in the southern, western, and eastern horn of the AP (along the Oman-UAE border), signifying a noticeable shift from the “no thermal stress” to the “moderate heat stress” category. In contrast, the remainder of the AP displays a declining trend, indicating a progression toward higher thermal stress categories. The number of “strong heat stress” days exhibits a visible pattern of increase (15–18 days/decade), with major hotspots in the southwestern and eastern parts of the AP (Fig. 4j–l). However, this upward trend is more prominent in UTCI_mean_ than in UTCI_min_ and UTCI_max_. Importantly, these regions encompass major metropolitan areas, including Jeddah, Madinah, Makkah, NEOM (a newly developing city in western Saudi Arabia), Dammam, and Muscat. Our results further demonstrate that the frequency of “strong heat stress” days, based on UTCI_max_ and UTCI_mean_, is significantly on the rise in the northern and southeastern regions of the AP at a rate of 3–15 days/decade (Fig. 4n,o), with the most substantial trend observed in the inland areas along the Saudi-Oman-Yemen border junction. It is worth noting that “very strong heat stress” days based on UTCI_min_ did not exhibit any discernible trend (Fig. 4m), owing to their lower intensity that does not surpass the recommended thermal thresholds in the study region. Furthermore, our findings reveal that when considering UTCI_max_, the frequency of “extreme thermal stress” days exhibits a general increase across the AP (Fig. 4q), with the most pronounced tendency (3–11 days/decade) observed in the southwestern, central, and eastern regions. These areas encompass major cities, including Jeddah, Makkah, Madinah, NEOM, Jizan, Riyadh, Dammam, Al-Hudaydah, Sanaa, Doha, and Al Ahmadi. Conversely, UTCI_min_ and UTCI_mean_ do not indicate any occurrences of “extreme heat stress” days (Fig. 4p,r), which can be attributed to their lower intensities observed in the AP, falling below the recommended thermal thresholds.

**Figure 5:**
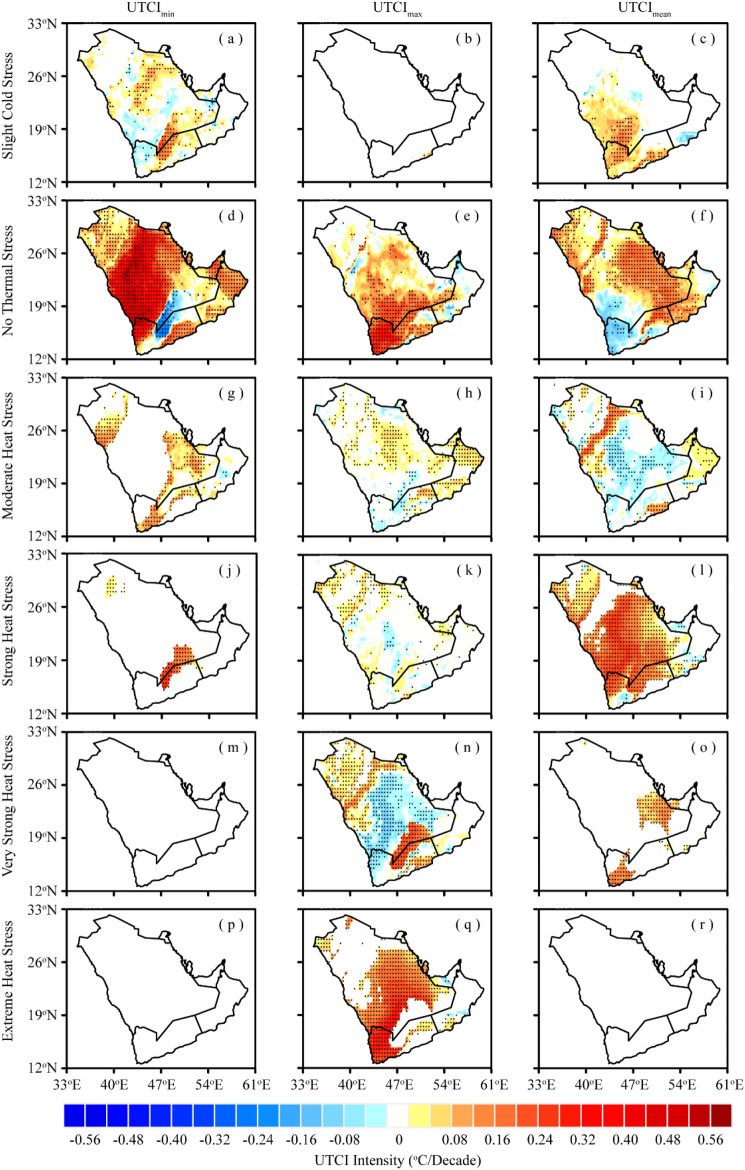
Spatial trends in the intensity of UTCI stress categories over AP during 1979–2022; (**a**–**c**) Slight cold stress, (**d**–**f**), No thermal stress, (**g**–**i**) Moderate heat stress, (**j**–**l**), Strong heat stress, (**m**–**o**) Very strong heat stress, and (**p**–**r**) Extreme heat stress. The black dots indicate that the trend is statistically significant at the 0.05 significance level (This fgure was created in GrADS 2.2.1).

Figure 5 illustrates the spatial distribution of trends in the intensity of UTCI categories over the AP region. Both UTCI_min_ and UTCI_mean_ exhibit an asymmetric pattern of intensity for the “slight cold stress” category, with the most significant increase (0.01–0.20 °C/decade) observed in the central, eastern, southern (Fig. 5a), and southwestern parts of AP (Fig. 5c). This signifies that these regions of AP have experienced a rise in the intensity of “slight cold stress” during the study period. In contrast, UTCI_max_ did not exhibit “slight cold stress” intensity due to higher daily maximum temperatures and lower thresholds for the “slight cold stress” category (Fig. 5b). The increasing tendency of the “no thermal stress” intensity is most pronounced (0.08–0.60 °C/decade) over most of the AP region (Fig. 5d–f), indicating normal climatic conditions during the study period. Some parts of the AP, such as the inland southwestern region along the Saudi–Yemen border have witnessed a decrease in the intensity of the “no thermal stress” category; however, in the case of UTCI_max_ and UTCI_mean_, this decreasing trend shifts to the northwestern, eastern, and southern parts of the peninsula.

Moreover, the intensity of the “moderate heat stress” category exhibits a mixed pattern of increasing and decreasing trends across the AP region (Fig. 5g–i). The UTCI_min_-based “moderate heat stress” intensity displays a dipole pattern with an increasing trend of 0.01–0.12 °C/decade in the northwestern and southwestern parts of AP. Conversely, the UTCI_max_-based intensity, while slightly reduced (0.01–0.04 °C/decade), extends over the central and extreme northeastern parts of the peninsula. Notably, the UTCI_mean_-based “moderate heat stress” intensity reveals a more evident decreasing trend; however, the upper central part of Saudi Arabia and the eastern horn of AP—major parts of Oman and UAE—experienced an increase of 0.01–0.20 °C/decade. The regions with increasing trends in “moderate heat stress” intensity encompass several major urban clusters within the AP, such as Jeddah, Makkah, Riyadh, Dammam, Muscat, Abu Dhabi, and Dubai, underscoring the extent of population exposure to extreme and persistent heat stress in these cities.

The results of all UTCI indices show a positive trend in the intensity of “strong heat stress” over the AP, with the maximum spatial extent in UTCI_mean_, followed by UTCI_max_, and UTCI_min_ (Fig. 5j–l). Both UTCI_min_ and UTCI_max_ show an increasing trend of 0.01–0.24 °C/decade in the northern and southern parts. However, the magnitude of this increasing trend is more pronounced in UTCI_min_, while its spatial extent is greater in UTCI_max_. In contrast, the intensity of the UTCI_mean_-based “strong heat stress” exhibits the highest increasing trend over major parts of AP; however, this increasing trend is strongly concentrated (0.12–0.28 °C/decade) in the central AP, indicating a significant presence of thermal discomfort in these areas. Additionally, the intensity of “very strong heat stress” based on the UTCI_max_ and UTCI_mean_ indices revealed a rising trend (0.01–0.24 °C/decade) in the western, southern, and eastern parts of the peninsula (Fig. 5n,o). However, the UTCI_min_-based intensity of “very strong heat stress” does not exhibit any significant trend (Fig. 5m), likely due to lower values of daily UTCI_min_, falling below the recommended thresholds for the “very strong heat stress” category. Furthermore, based on UTCI_max_ (Fig. 5q), a significant increase is observed in the intensity of “extreme heat stress” over major parts of the AP, with substantial intensification (0.16–0.44 °C/decade) in the major urban clusters, including Jeddah, Makkah, Jizan, Riyadh, Dammam, Al-Hudaydah, Sanaa, and Doha. In contrast, the UTCI_min_ and UTCI_mean_ indices do not signify any “extreme heat stress” magnitude (Fig. 5p,r), due to their lower intensities falling below the recommended thermal thresholds.

**Figure 6:**
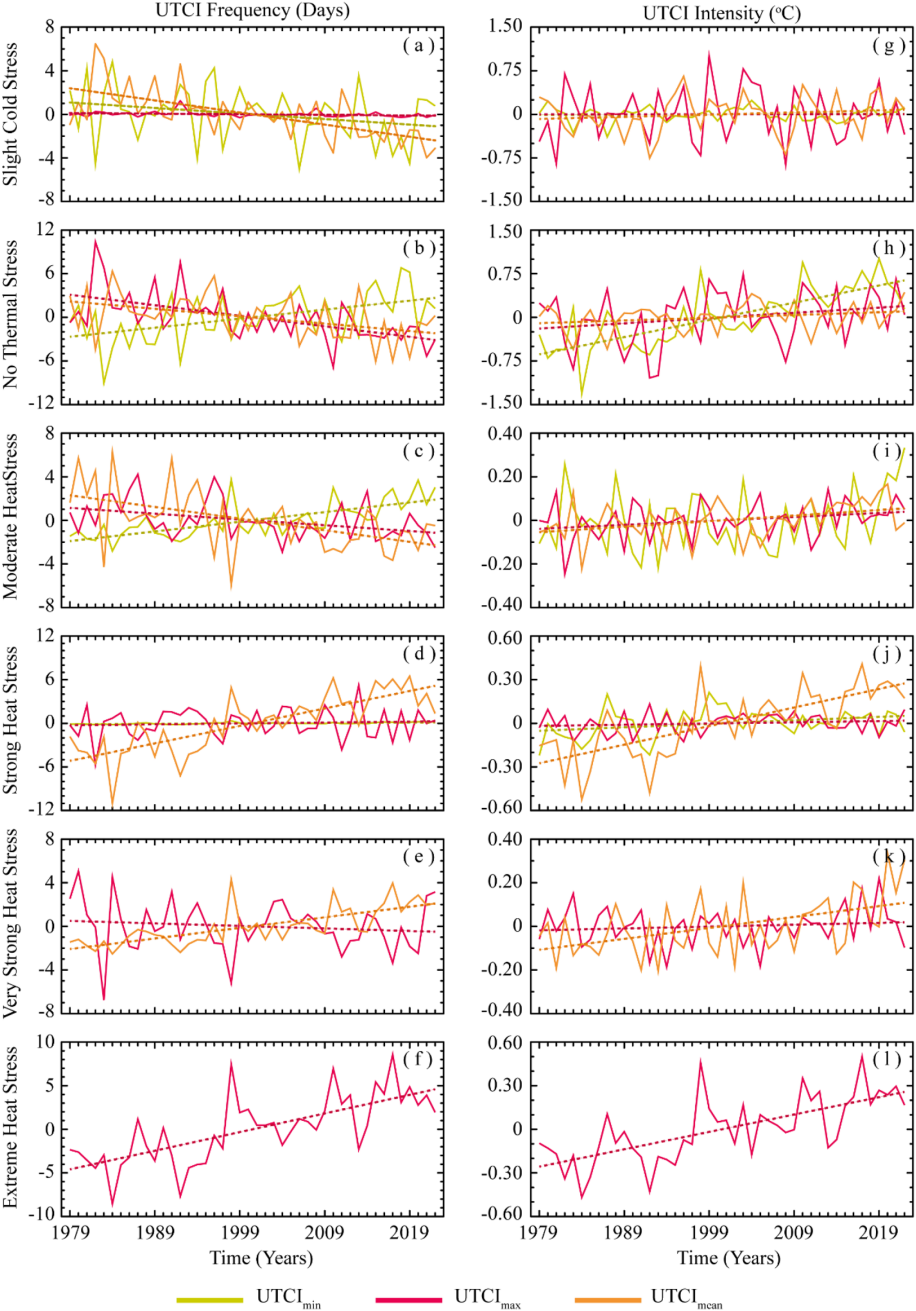
Temporal trends in the frequency and intensity of UTCI stress categories over AP during 1979–2022; (**a**–**f**) frequency and (**g**–**l**) intensity of UTCI indices. The solid lines in different colors indicate the annual anomalies of UTCI indices, while the dotted lines indicate their respective linear trends (This fgure was created in Matlab R2022b).

Figure 6 depicts the temporal patterns of annual anomalies and linear trends in the frequency and intensity of UTCI stress classes over AP during 1979–2022. In terms of frequency, the number of days with “slight cold stress”, “no thermal stress”, and “moderate heat stress” decreased (Fig. 6a–c), while those of “strong heat stress”, “very strong heat stress”, and “extreme heat stress” increased significantly during the study period (Fig. 6d–f). Irrespective of their counterparts, the frequency of UTCI_max_-based “very strong heat stress” days decreased (Fig. 6e), while the number of days with “no thermal stress” and “moderate heat stress” based on UTCI_min_ increased during the study period (Fig. 6b,c). The temporal distribution of annual anomalies reveals a sharp increase in the frequency of human thermal stress during 1998 and onward, indicating a shift towards a hotter climate for a prolonged period in the study region. As shown in Table 2, the outcomes of the monotonic trend indicate that the number of “slight cold stress”, “no thermal stress”, and “moderate heat stress days” has decreased in the range of − 0.564 to − 1.040, − 1.091 to − 1.133, and − 0.491 to − 1.014 days/decade, respectively. Whereas the frequency of “strong heat stress”, “very strong heat stress”, and “extreme heat stress” has increased at the rates of 0.052–2.334, and 2.072 days/decade, respectively.

**Table 2:** Monotonic temporal trend in the frequency of UTCI stress categories.

Stress category	Frequency (days/decade)		
	UTCI_min_	UTCI_max_	UTCI_mean_
Slight cold stress	− 0.564	− **0.052**	− **1.040**
No thermal stress	**1.203**	− **1.133**	− **1.091**
Moderate heat stress	**0.832**	− **0.491**	− **1.014**
Strong heat stress	**0.064**	0.052	**2.334**
Very strong heat stress	–	− 0.365	**0.921**
Extreme heat stress	–	**2.072**	–

The bold values indicate that the trend is statistically significant at the 0.05 significance level.

In terms of intensity (Fig. 6g–l), most of the stress categories exhibited an increasing trend, suggesting that the peninsula has been dominated by significant thermal discomfort during the study period. The temporal pattern of annual anomalies shows that the intensity of thermal heat stress classes sharply increased in the later years of the period. The increasing intensity of the thermal stress categories indicates the consistent occurrence of intense heat stress in the study region. A suite of the literature revealed that the major parts of AP have witnessed several episodes of heat extremes over the past few decades^63,76,77^, affirming the findings of this study. Given the monotonic trend (Table 3), the intensity of the “slight cold stress”, “no thermal stress”, “moderate heat stress”, “strong heat stress”, “very strong heat stress”, and “extreme heat stress” has increased at the rates of 0.009–0.032, 0.037–0.298, 0.016–0.028, 0.022–0.120, 0.003–0.050, and 0.117 °C/decade, respectively, indicating their evolution into higher stress categories with consistent and considerable thermal stress.

**Table 3:** Monotonic temporal trend in the intensity of UTCI stress categories.

Stress category	Intensity (°C/decade)		
	UTCI_min_	UTCI_max_	UTCI_mean_
Slight cold stress	− 0.002	0.009	0.032
No thermal stress	**0.298**	0.078	0.037
Moderate heat stress	0.026	0.016	**0.028**
Strong heat stress	0.022	0.008	**0.120**
Very strong heat stress	–	0.003	**0.050**
Extreme heat stress	–	**0.117**	–

The bold values indicate that the trend is statistically significant at the 0.05 significance level.

### Role of ENSO on UTCI Amplification

**Figure 7:**
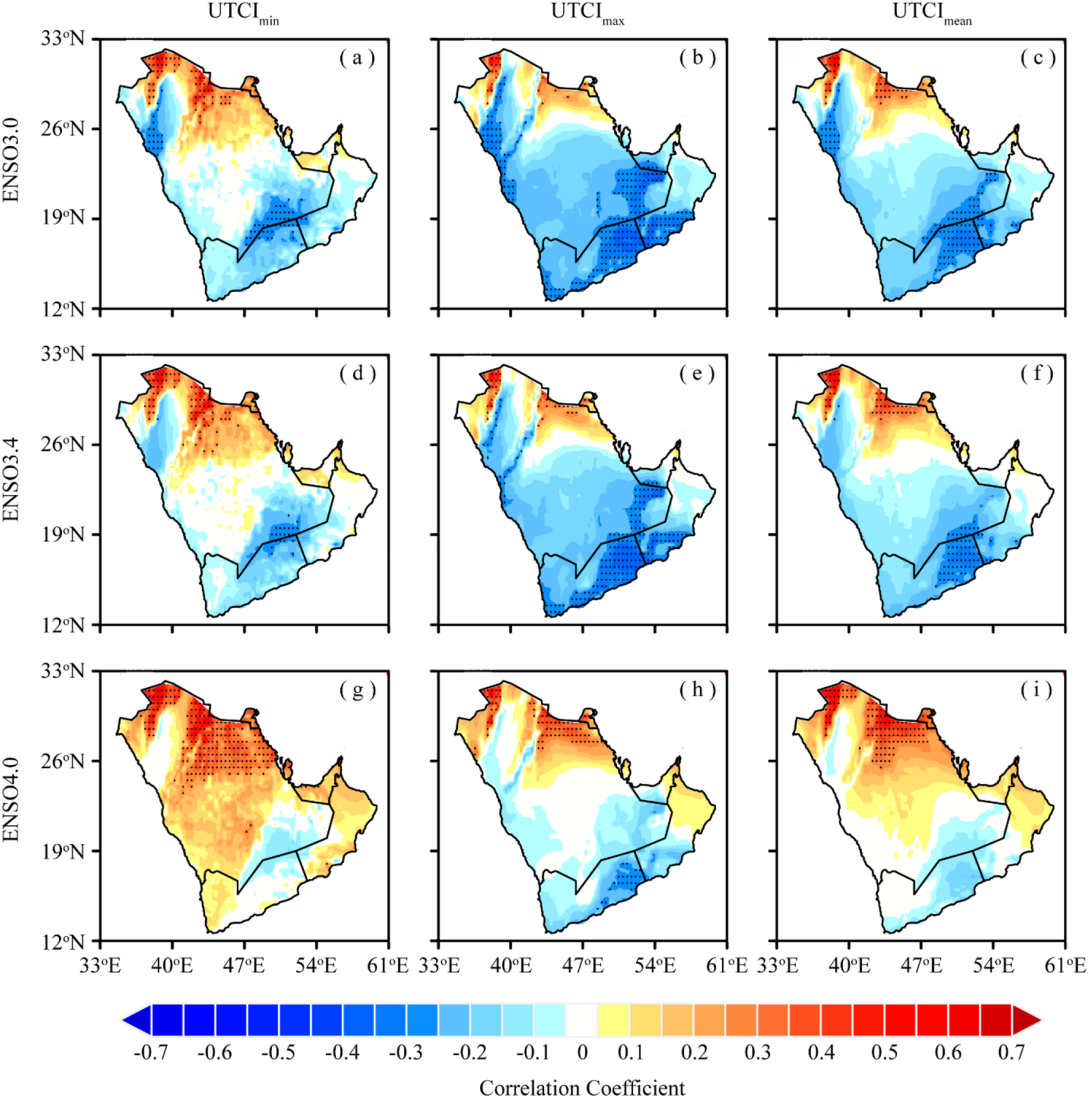
Spatial correlation between UTCI and ENSO indices over AP during 1979–2022; (**a**–**c**) ENSO3.0, (**d**–**f**) ENSO3.4, and (**g**–**i**) ENSO4.0. The black dots indicate that the correlation is statistically significant at the 0.05 significance level (This fgure was created in GrADS 2.2.1).

Figure 7 shows the spatial correlation between the annual mean of daily UTCI_min_, UTCI_max_, and UTCI_mean_ and three ENSO indices, including ENSO3.0, ENSO3.4, and ENSO4, representing the Pacific Ocean surface temperature in the eastern, central, and western parts of the Pacific during 1979–2022, respectively. The correlation between the UTCI_min_ and the three ENSO indices shows a dipole pattern with negative (< − 0.50) values in the eastern AP and positive values (0.60) in the southwestern parts. The UTCI_max_ and the ENSO indices show a similar correlation pattern, but the geographical spread of the negative correlation shows that the eastern Pacific surface temperature (ENSO3.0 and ENSO3.4) has widespread effects on the UTCI_max_ than the western Pacific Ocean surface temperature where a strong dipole is evident. The UTCI_mean_ shows a similar correlation as observed for UTCI_max_ with ENSO indices, except for the ENSO4.0, which has a significant effect on the dipole pattern of this correlation. The ENSO and UTCI indices relationship over the AP shows two distinct patterns, including the dipole effect of the western Pacific forcing (ENSO4..0) which is more prominent for the UTCI_min_ than for the UTCI_max_ and UTCI_mean_. The eastern Pacific ENSO indices (ENSO3.0 and ENSO3.4), on the other hand, have a significant negative relationship with UTCI_max_ and UTCI_mean_ values, implying that a positive mode of the ENSO in the eastern Pacific would produce below-normal human thermal stress and vice versa. The same is evident for the ENSO4.0 but the dipole pattern shows a regionally varying effect.

## Discussion

Global warming-induced climate change coupled with population expansion and rapid urbanization has amplified human thermal stress in urban environments, which could adversely affect the comfort level of the local population. This study aims to assess spatiotemporal changes in human thermal stress over the AP region and its urban centers using multiple UTCI indices for the period 1979–2022. The results of the study show that the southeastern and southwestern parts of AP have higher climatological values of UTCI indices, suggesting that these parts have experienced considerable thermal stress in recent decades. This further highlights the level of vulnerability of these regions to climate change and its heat extremes. The higher intensity of UTCI indices in these parts of AP can be attributed to their distinct location, complex climate, and diverse topography, as the southeastern region has a hyper-arid climate with desert topography, high temperatures, and limited precipitation^63^, while the southwestern region is located along the coast of Red Sea, having hot-humid climate and complex topographic features. On the other hand, the northern and northwestern parts of AP have the lowest climatological mean of UTCI indices, which can be linked to the onset of the cold Siberian High, the passage of the Mediterranean cyclones and the fronts, and the formation of the Shamal winds^78^.

In terms of the annual cycle, the study region has the warmest thermal conditions in July and August, followed by June, September, and May, while the coldest thermal conditions are in January and February, followed by March, and December. These results indicate that the AP region is under the strong (weak) influence of thermal stress during the summer (winter) months. The results closely align with the findings of recent studies^13,37,79^, which reported similar warm (cold) thermal conditions during the summer (winter) months in different parts of the world. It is worth mentioning that both the warm and cold thermal months reflect the summer and winter seasons of the AP region, where the summers are extremely hot, while the winters are relatively mild. During the summer season, the AP climate is often hot-humid—daytime temperatures sometimes exceed 50 °C with high humidity, while in winters, the climate is relatively milder with cold breezes at nighttime—temperatures remain less than 20 °C with the least heat stress.

In terms of the long-term trend, most of the AP parts, including the southwestern, central, northeastern, and southeastern parts have experienced a significant increase in UTCI indices, suggesting pronounced thermal stress in these regions during the study period. It is worth mentioning that these parts of AP comprised some of the major global urban centers. This increasing trend of UTCI indices can be attributed to various natural and anthropogenic factors, including geographical features, land-sea interactions, topographical influences, population expansion, rapid urbanization, and urban heat island effects^5,63,80,81^. Moreover, the UTCI indices exhibited an asymmetric warming trend in the AP, with a higher magnitude and larger spatial extent in UTCI_min_, followed by UTCI_mean_ and UTCI_max_, which can be attributed to the sharp increase in daily minimum temperature to maximum and mean temperatures and their related extremes^64,77,81^. Such heterogeneous patterns of minimum, maximum, and mean temperatures have been observed in AP and other parts of the world, which confirm our current findings^13,31,32,58,59^.

Furthermore, the long-term temporal anomalies exhibit a linear increase during the study period, with a sharp increase during the late 1990s and onward, which indicates a shift toward a more intensified regional climate and pronounced heat stress in recent decades in AP. Similar results are reported by several studies, stating that over the last two decades, many parts of the AP region have experienced the highest surface temperature and intense heat extremes, with increased health-related risks^63,82^. The World Meteorological Organization (WMO) also declares that the last two decades (2001–2020) as the hottest ones in the world's history^83,84^. The warming trend in UTCI indices and resultant thermal stress in the urban centers of AP is consistent with broader global climate change trends, which may have implications for local human populations. These findings are of paramount importance as they provide a comprehensive overview of the spatial distribution and increasing trend of thermal stress in AP, highlighting regions of particular concern for diverting climate change adaptation and mitigation measures.

We further studied the frequency and intensity of the UTCI categories and revealed the southwestern, northern, and central parts of the AP experienced a significant increase in the frequency and intensity of “strong heat stress”, “very strong heat stress”, and “extreme heat stress” categories. Importantly, these regions encompass major metropolitan areas, including Jeddah, Madinah, Makkah, NEOM, Dammam, Muscat, Jizan, Riyadh, Al-Hudaydah, Sanaa, Doha, Al Ahmadi, and the inland areas along the Saudi-Oman-Yemen border junction. The increasing frequency and intensity of thermal stress implies that these cities have experienced frequent and intense episodes of extreme heat stress during the study period. This suggests that the urban population in the AP has experienced frequent and intense heat stress, potentially leading to heat-related health issues, such as heat exhaustion and heat stroke^49,50,76^. Similar to UTCI indices, the temporal distribution of annual anomalies of UTCI stress categories reveals a sharp increase in the frequency and intensity of human thermal stress during 1998 and onward, indicating a shift towards a persistent hot climate in the peninsula during the study period, which could have adverse impacts on the comfort level of the local population. Recently, several studies reported similar hot climate patterns, with substantial impacts on the local population in the peninsula and neighboring regions^50,85–87^.

Overall, the rise in the frequency and intensity of extreme thermal stress in the AP can be attributed to its distinct geographical location, multifaceted topography, complex climatic conditions, regional and global atmospheric systems, urban development, and population growth^63,88^. It is important to mention that the cities located in the southwestern and eastern parts of the AP have hot-humid climates due to their proximity to the Red Sea and the Arabian Sea, which acts as a major source of moisture and water vapor for humid weather in the coastal areas^89,90^. The increase in the number and magnitude of heat stress episodes in hot-humid cities is also driven by rapid urbanization and population expansion, particularly in the AP region^63,91^. Whereas the southern cities of Jizan, Al-Hudaydah, and Sanaa are located at higher altitudes, the increasing number of heat stress days in these cities can be linked to elevation-dependent warming—a phenomenon in which high altitude areas experience relatively higher warming than those of the low-lying plains^92,93^. The central and inland cities have a typical arid-hot climate and are located in or near the Rub' al Khali, which is an integral part of the Arabian desert. Consequently, policymakers and city planners should consider these trends when formulating strategies to mitigate the adverse impacts of heat stress on the local population in AP and other regions with similar climatic characteristics.

The ENSO and UTCI indices relationship over the AP shows two distinct patterns, including the dipole effect of the western Pacific forcing (ENSO4.0), which is more prominent for the UTCI_min_ than for the UTCI_max_ and UTCI_mean_. The eastern Pacific ENSO indices (ENSO3.0 and ENSO3.4), on the other hand, have a significant negative relationship with UTCI_max_ and UTCI_mean_ values, implying that a positive mode of the ENSO in the eastern Pacific would produce below-normal human thermal stress and vice versa in the AP region. Similar results were previously reported for temperature anomalies as a consequence of the ENSO-induced circumglobal changes in the circulations^1,2^. The same is evident for the ENSO4.0 but the dipole pattern shows a regionally varying effect. The mechanism of the dipole pattern for the eastern Pacific and UTCI_min_ could be related to the ENSO-driven monsoon circulation that affects the South Asian monsoon through controlling the zonal easterlies and hence a negative relationship with the eastern parts of AP and positive relationship with the western AP^72,73,94,95^. The eastern Pacific on the other hand appeared to have widespread global effects and thus its positive mode could affect the Indian Ocean basin-wide forcing and the overall thermal stress regime of the AP region^96–99^.

## Conclusion

The study analyzed spatiotemporal changes in human thermal stress categories and their characteristics and investigated the correlation between the UTCI and ENSO indices in the AP during the period 1979–2022. The results of the study show that the major urban centers in the southwestern and central parts of the AP have experienced a significant increase in UTCI-based human thermal stress, indicating the predominance of frequent, intense, and prolonged human thermal discomfort during the study period. These urban centers include Jeddah, Jizan, Makkah, Madinah, Riyadh, Abha, NEOM, Riyadh, Dammam, Abu Dhabi, Dubai, Doha, Muscat, Al-Hudaydah, and Sanaa. This increase in the frequency and intensity of thermal discomfort in AP's urban centers can be attributed to their distinct geographical locations, complex climatic conditions, regional and global atmospheric systems, urbanization, and population growth. The results further demonstrate a consistent increase in human thermal stress and its frequency and intensity over the years, particularly from 1998 onwards, indicating a transition towards a hotter climate with frequent and intense heat extremes for a prolonged period in the study region. The correlation between the UTCI and ENSO indices shows a dipole pattern with a positive pattern in the southwestern and a negative pattern in the eastern AP, which modulates the thermal stress regime in AP. The ENSO evolution in the eastern equatorial Pacific (ENSO3.0 and ENSO3.4) significantly affects the UTCI_max_ and UTCI_min_ whereas the western Pacific (ENSO4.0) has significant control over the UTCI_mean_ pattern in a much larger area compared to the eastern Pacific indices. The results of the study call for particular attention to public health and well-being in urban areas, particularly vulnerable groups, such as the elderly, children, women, disabled individuals, laborers, and marginalized populations. The findings emphasized the need for policymakers to consider these trends for effective climate change adaptation and mitigation strategies to address the growing impact of heat stress on the AP's urban population. Furthermore, the results urged that city and urban planners need to carefully strategize and develop future cities and towns in areas, which are less vulnerable to the adverse effects of climate change, particularly heat stress.

## Data Availability

The datasets used in this study are publicly available at their given links. The ERA5-HEAT data can be obtained from the Copernicus Climate Data Store (CDS) website of the Copernicus Climate Change Service (C3S) and the European Centre for Medium-Range Weather Forecasts (ECMWF) (https://cds.climate.copernicus.eu/cdsapp#!/dataset/derived-utci-historical?tab=overview). The ENSO indices time series data can be accessed from the National Center for Atmospheric Research (NCAR) website (https://psl.noaa.gov/data/climateindices/list/).

## Code availability

The codes used for data processing can be provided on request to the corresponding author.
